# Improving management of needle distress during the journey to dialysis through psychological education and training—the INJECT study feasibility pilot protocol

**DOI:** 10.1186/s40814-022-00989-2

**Published:** 2022-02-04

**Authors:** G. Radisic, E. Duncanson, R. Le Leu, K. L. Collins, A. L. J. Burke, J. K. Turner, A. Chur-Hansen, F. Donnelly, K. Hill, S. McDonald, L. Macauley, S. Jesudason

**Affiliations:** 1 Central Northern Adelaide Renal and Transplantation Service, Royal Adelaide Hospital, Adelaide, South Australia 5000 Australia; 2 School of Psychology, Faculty of Health and Medical Sciences, University of Adelaide, Adelaide, South Australia 5000 Australia; 3 School of Medicine, Faculty of Health and Medical Sciences, University of Adelaide, Adelaide, South Australia 5000 Australia; 4 Psychology Department, Royal Adelaide Hospital, Adelaide, South Australia 5000 Australia; 5 University of South Australia, South Australia 5000 Adelaide, Australia

**Keywords:** Haemodialysis, Needle distress, Psychology, Education, Training

## Abstract

**Background:**

Needle-related distress is a common yet poorly recognised and managed problem among haemodialysis (HD) patients. The aim of this pilot study is to test the feasibility and acceptability of the INJECT Intervention—an innovative psychology-based intervention to empower patients to self-manage needle distress with the support of dialysis nurses.

**Methods:**

This investigator-initiated, single-arm, non-randomised feasibility study will take place in a large dialysis service in Adelaide, Australia. Participants will include patients aged ≥ 18 years, commencing or already receiving maintenance HD, recruited through dialysis physicians and nursing staff as individuals believed to be at risk of needle distress. They will be screened for inclusion using the Dialysis Fear of Injection Questionnaire (DFIQ) and enrolled into the study if the score is ≥ 2. The multi-pronged intervention encompasses (i) psychologist review, (ii) patient self-management program and (iii) nursing education program. The primary aim is to evaluate feasibility and acceptability of the intervention from patient and dialysis nurse perspectives, including recruitment, retention, engagement with the intervention and completion. Secondary exploratory outcomes will assess suitability of various tools for measuring needle distress, evaluate acceptability of the nursing education program and measure cannulation-related trauma and vascular access outcomes.

**Conclusion:**

The results will inform the protocol for larger trials addressing needle distress in HD patients.

**Trial registration:**

Australian New Zealand Clinical Trials Registry (ANZCTR): ACTRN12621000229875, approved 4 April 2021, https://www.anzctr.org.au/.

**Supplementary Information:**

The online version contains supplementary material available at 10.1186/s40814-022-00989-2.

## Introduction

Kidney failure is a complex debilitating chronic illness. Severe kidney failure requires kidney replacement therapy (dialysis or kidney transplantation) to sustain life. Although dialysis prolongs life, it has a major effect on patients’ medical, social and psychological wellbeing as they are tied to their treatment 3–7 days per week. In Australia in 2019, approximately 14,000 people received dialysis, 83% of which were treated with haemodialysis (HD) [1].

One of the challenges for haemodialysis patients is frequent and repeated exposure to needles over a long period. Needle-related distress is common amongst general healthcare users [2] and given that the average patient receiving haemodialysis has 2 large bore needles inserted three times per week (312 per year), it is not surprising that needle distress constitutes a significant treatment barrier or burden for many patients [3]. Fear of needles can influence patients’ treatment choice [4, 5] and even result in refusal of treatment [6–8]. Needle distress is often underreported by patients and not routinely assessed by dialysis staff [9, 10]. There are different terms used to describe negative emotional responses that are associated with needle procedures; however, here we use the term needle distress to describe stress, fear and anxiety associated with needle-related procedures.

The *I*mproving Management of *N*eedle Distress during the *J*ourney to Dialysis through Psychological *E*du*C*ation and *T*raining (INJECT) study was informed by findings from several other studies performed by a clinical research group within our local health service. In a point prevalence survey of 551 dialysis patients in a large South Australian dialysis service completed in 2018, 36% of patients reported needle fear, of whom 37% reported that this influenced their treatment choices [3]. In another observational study examining outcomes of vascular access within 6 weeks of starting HD within two South Australian hospitals (*n* = 117), 54% of patients experienced difficulty with insertion of needles [11]. Difficult cannulations are considered a likely contributor in developing needle fear [12] while vascular access has been flagged as a core outcome of critical interest for patients receiving dialysis and other clinical stakeholders [13]. Vascular access complications are one of the major reasons for hospitalisations of patients on HD and are linked with increased mortality, morbidity and cost [13].

We also conducted qualitative interviews with the local health service HD patients (*n* = 15) and nurses (*n* = 17) to explore their experiences, perceptions and opinions regarding needle fear. Both patients and nurses agreed that cannulation can be a traumatic process and that needle fear can directly influence patients’ treatment preferences and engagement, as well as their mental health and quality of life. Both groups also reported a lack of knowledge about needle distress and ways to manage it, emphasising that needle distress is not routinely recognised, validated, assessed or addressed in HD treatment. They also highlighted the critical role of nurses to successful cannulation process and minimising distress. These interviews allowed for the identification of processes involved in preventing, minimising and exacerbating needle fear and distress, including potential management strategies and supports. Data obtained from these interviews informed the development of this pilot patient-led and nurse-supported intervention to address needle fear.

Although studies have investigated alleviating needle distress in non-dialysis settings, few have been conducted in clinical settings [14]. In particular, there are no tested interventions to proactively managing needle distress for those who require longer-term dialysis [10] To address this gap in care, we have developed an educational program based on the principles of cognitive–behavioural therapy (CBT). This program is designed to educate both HD patients and nurses about needle distress and to provide self-management strategies patients can apply to manage fear of dialysis needles.

CBT is a gold standard therapy for anxiety disorders [15–17] and psychological distress [18, 19]. CBT-based strategies have been successfully utilised in a self-help context for a range of mental and physical difficulties [20, 21]. Its basic premise is that our perception of a situation determines how we feel and respond, rather than the situation itself, and that the way we behave can impact the way we feel and vice versa. Thus, in order to reduce distress or unhelpful behaviour, the thoughts that are driving those feelings and behaviours must be identified and challenged. CBT can be delivered face-to-face or digitally; however, the past decade has seen a rapid increase in the digital delivery of CBT interventions. This mode of delivery is a low-intensity, guided self-help [22] that is equivalent in its effectiveness to face-to-face interventions [15, 23].

**Fig. 1:**
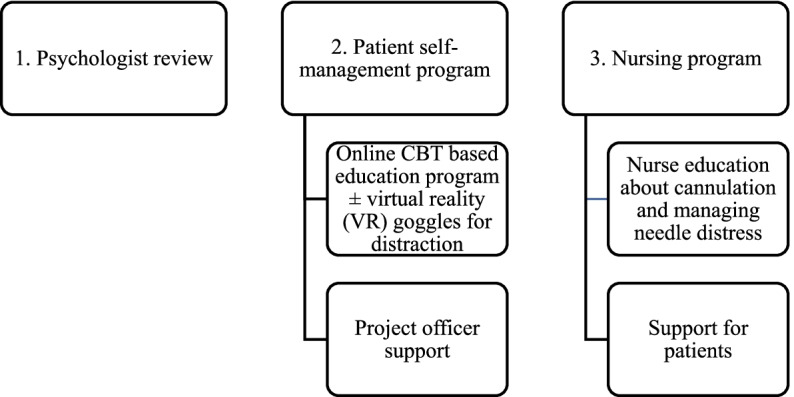
Intervention components

The educational program is one component of the “*I*mproving Management of *N*eedle Distress during the *J*ourney to Dialysis through Psychological *E*du*C*ation and *T*raining” (INJECT) intervention. The multicomponent intervention (Fig. 1) incorporates (1) psychologist review, (2) patient self-management program and (3) nursing program.

The INJECT intervention provides an option to use VR technology as a distraction tool during cannulation. Distraction can aid the management of fear and pain as it diverts a patient’s attention away from unpleasant procedures [24]. Recently, there has been an increase in the number of studies using VR to manage distress and pain associated with needle procedures and chronic conditions [25]. However, the majority of these studies have focused on children and adolescents, while studies involving adults remain limited [25–28]. A recent scoping review explored the use of VR and its effect on the level of engagement in self-care and health-related quality of life of adult patients receiving HD, concluding that it had a positive impact on physical and mental health [29].

The INJECT study is an innovative intervention that aims to better identify needle distress among dialysis patients as part of routine care and empower patients to self-manage needle distress with the support of dialysis nurses. Patients are encouraged to be active participants in their own wellbeing through this opportunity to learn skills to overcome fear of needles. Nurses can improve care delivery by providing support to patients in applying learned strategies to manage needle distress. The goal of this pilot is to assess feasibility and acceptability of each component of the intervention involving patients and dialysis nurses and to use study findings as a guide in the design and implementation of a larger-scale study. We believe that the intervention has a potential to be transferred to other chronic disease patient groups who experience high needle burden as part of treatment, such as receiving chemotherapy.

## Methods

### Study design

The INJECT study is a single-arm, open-label, non-randomised feasibility pilot that will assess a multifaceted intervention to improve needle distress in HD patients. The study intervention components are shown in Fig. 1:

1. Psychologist review (an overall psychologist assessment, completion of baseline questionnaire and explanation of CBT principles + introduction to VR)
2. Patient self-management program (online CBT education modules with an option to use VR as distraction and support from the research officer)
3. Nursing program (nurse education about needle distress and ways to support patients with their self-management).

**Fig. 2:**
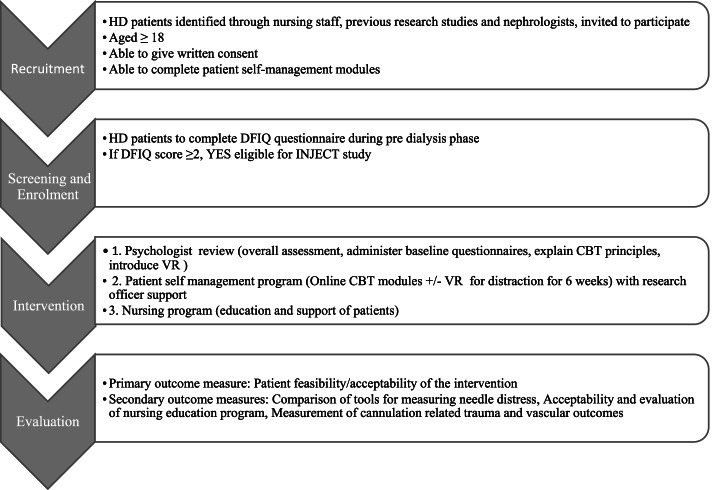
Study flow

A suite of novel strategies forms this entire intervention. Each of the components was chosen and developed based on extensive nursing and patient input and will respectively be tested and evaluated. No control group will be employed in this pilot study because there are no inferential comparisons to be conducted. The study flow is presented in Fig. 2.

This study follows the SPIRIT 2013 [30] guidelines and reporting template (Additional file 1) and CONSORT 2010 statement: extension to randomised pilot and feasibility trials [31] (Additional file 2).

### Participants

HD patients will be recruited from a large South Australian dialysis service. The study will be conducted at 3 metropolitan sites (2 based in hospitals and 1 satellite unit). Inclusion/exclusion criteria are presented in Table 1 below.

**Table 1:** Inclusion/exclusion criteria

~ 25 Haemodialysis (HD) patients	
Inclusion	Exclusion
Step 1	
• > 18 years • English speaking • Commencing HD within study timeframe (incident patients) • Already receiving HD • Current AV fistula or graft being used for cannulation • In-patient or outpatient	• Unable to give written consent • Currently receiving psychological therapy/intervention for needle fear/distress • Inability to complete patient self-management modules (e.g. due to literacy, vision impairment, cognitive deficits) • No current exposure to dialysis needles • DFIQ < 2
Step 2	
• HD patients: DFIQ score ≥ 2	

Patients with kidney failure currently receiving, or about to commence, HD will be eligible to participate in the study. The study will be advertised to patients in dialysis units by nurses. Nephrologists and nurses can also promote the study to patients who they suspect may have needle fear or distress and invite them to participate. Nephrologists and nurses will use their clinical judgement and patient review to identify patients that may have the fear of needles. HD nurse practitioner will promote the study to all new haemodialysis patients. The research officer will follow up with the patients and provide them with the patient information sheet and consent form. Following receipt of written informed consent, patients will be screened for inclusion with a validated measure of needle distress: Dialysis Fear of Injection Questionnaire (DFIQ) [9]. Those who score ≥ 2 will be eligible for participation.

This study will be open for recruitment from March 2021 to September 2021.

### Study intervention

The intent of the intervention is not to eradicate distress, but rather to equip patients with skills to more effectively manage distress related to needle fear. The intervention has been co-designed by a multidisciplinary team comprised of researchers, medical professionals, psychologists, nurses and consumer partners. The design of the intervention is flexible and adaptable, and as it does not necessarily require face-to-face contact, it can easily be modified to full online application.

The intervention will have a strong emphasis on skill acquisition and practice, and it will encompass following three components:

- *Psychologist review*—Following enrolment, patients will receive a 90-min consultation with a clinical psychologist who will be a coinvestigator of the study. This will include an overall psychological assessment, suggestion for ongoing clinical care or referral (if required), completion of baseline questionnaires, provision of an overview of the CBT self-management modules, a demonstration of the VR set and option to test it.

- *Patient self-management program*—Psychoeducation about needle distress and strategies that patients can apply to manage this will be delivered as a short course in an online learning platform. The course will consist of 6 modules with each module taking 10 to 15 min to complete. The modules were developed by the investigator team with consumer input and consist of videos and written materials explaining CBT principles and strategies tailored to the dialysis needle fear context. The key content summary of learning outcomes is presented in Table 2. Patients will be advised that the online program should be completed within 6 weeks. Although they will be encouraged to progress through the modules at the rate of one module per week, participants can choose to complete the modules at a faster rate if they wish to do so. Patients will be emailed the link to the course which can be accessed on any internet-enabled device such as a computer, laptop, tablet or mobile phone. Modules can be completed during dialysis sessions or at other times suitable to participants. Electronic devices (tablets) will be available for patients to use during dialysis sessions. Paper versions of resources will also be available upon patient request to support the online content.

**Table 2:** Key content summary of the patient education program

Modules	Learning outcomes
1. Introduction to needle distress	− What is needle distress, the causes and how it can be experienced - Basic concepts of CBT (cognitive, emotional and behavioural signs of distress) - Ways people may cope with needle distress - Link between distress and avoidance − Self monitoring and why it is important
2. Relaxed breathing	- Rationale and evidence for relaxation - Practical exercise—the skill of relaxed breathing as a way of managing distress or anxiety − Tips for practising/using relaxation
3. Visual imagery	- The use of visual imagery for relaxation - Guided visual imagery exercise − Tips for practising/using visual imagery
4. Cognitive strategies	- How thoughts, beliefs and attitudes influence our experience of distress or anxiety - Guided mindfulness of thoughts exercise − Cognitive strategies for managing unhelpful thoughts
5. Managing difficult emotions	- Acceptance of distress and present moment awareness - Practical exercise: guided mindfulness of emotions - Helpful tips − The use of virtual reality (VR) technology as a distraction (immersion into different environment)
6. Summary	- Review of key strategies - Preparing for slip-ups/setbacks − Further support

Patients will have an option to use VR headsets (oculus Go) at dialysis, as distraction during cannulation if desired. The research team will have several sets with calming, relaxation videos such as ‘Calm on Oculus Go’ ready to be used. Patients will be instructed by the research officer to notify them directly, or via nurses, if they choose to use VR technology so that headsets can be provided.

The research officer will maintain regular weekly follow-ups with participants to track and ensure engagement and progression through the modules, remind participants to use CBT techniques ± VR, and to provide any other support patients may require during the project.

- *Nursing program*—The nurse education program aims to complement the patient self-management intervention. Nurses will complete the nurse education program prior to the enrolment of patients into the study and be available to support patients with the use of self-management strategies at dialysis. The education program consists of 2 modules that will be available on a local hospital online education platform. This program has been newly developed by the investigator team and teaches best practice techniques for cannulation with minimal trauma, as well as introducing nurses to the INJECT patient program content. Nurses will be taught how to best support patients’ use of self-management strategies during dialysis sessions. Once patients are enrolled in the INJECT intervention, nurses involved in their care will be provided with further education from the project officer, have the opportunity to revisit the online content, and will be given written prompts to refer to at each dialysis session to better support patients with needle distress.

### Data collection

At the enrolment, informed consent and demographic data will be collected. Demographic questionnaire is listed in Additional file 3. A schedule for enrolment, intervention and assessments is displayed as per SPIRIT guidelines in Table 3. Data collected prior to participant’s withdrawal will be used in de-identified form for group analysis and reporting. Course analytics from the online learning platform will enable tracking of patients’ learning and progress through the modules.

**Table 3:** Schedule of enrolment, intervention and assessments

	Study period			
	Enrolment	Baseline	3 weeks	6 weeks
		psychological assessment	Post education program completion	
**Timepoint**	**-** ***t***_**1**_	***t***_**0**_	***t***_**1**_	***t***_**2**_
**Enrolment:**				
Informed consent	X			
Eligibility screen- Dialysis Fear of Injection Questionnaire (DFIQ)	X			
Demographics	X			
**Intervention** (6-week duration starting from baseline)				
**Assessments:**				
DFIQ			X	X
Managing Needle Distress Questionnaire (MNDQ)		X		X
Blood/ Injection Fear Scale (BIFS)		X	X	X
Hospital Anxiety & Depression Scale (HADS)		X	X	X
Patient INJECT evaluation survey	At the end of 6-week intervention			
INJECT study satisfaction survey	Immediately post the completion of online education program			
Qualitative semi-structured interview study with nurses and patients	Following the completion of the pilot study			

### Qualitative study

At the end of the pilot, we will conduct semi-structured interviews with nurses and patients who participated in the study. Interviews will be conducted by a person not involved in study delivery. Interview questions will explore the acceptability of the intervention, feedback about the conduct of the study, preferences and perspectives on each element of the intervention, and barrier or facilitators to participation or completion. Interviews will be recorded, transcribed verbatim and thematically analysed by the research team using NVivo 12 software [32].

### Sample size

As this is a pilot feasibility study that does not propose inferential tests, power calculation is not essential. Our sample size calculation is based on the pragmatics of recruitment and the necessities for examining feasibility among a broad group of patients. We aim to recruit 25 patients. Allowing for a conservative 25% dropout due to death, illness, transplantation, change in dialysis modality and retention rates, we aim to have 20 patients in total.

### Outcome measures and evaluations

Outcome measures are presented in Table 4. We intend to undertake a thorough process evaluation of each component of the INJECT intervention from both a patient and nurse perspective to obtain a substantial body of feedback about acceptability and feasibility. We anticipate 50% retention rate for patients who are enrolled into the study. We acknowledge that undertaking a self-directed CBT program may not be of interest to all patients with needle fear. Based on previous studies reporting on the use of CBT interventions, dropout rates for psychotherapy range from 16% at the pretreatment phase to 26% at the treatment phase, with some studies reporting an average dropout rate of 47% [35, 36]. Acceptability will be assessed qualitatively, and we anticipate that the intervention will be acceptable from our qualitative approaches. We are unable to put a specific percentage on the reach in this intervention. In our point prevalence survey study, 36% of patients reported needle fear [3]; however, some of them may adapt and will not have an ongoing needle fear. We will use a range of quantitative and qualitative methods including tools to measure needle distress, evaluation surveys and direct interviews to evaluate the experience of patient and nurse participants. Self-devised Patient INJECT evaluation survey and INJECT study satisfaction survey will be pretested with the first 5 patients enrolled in the study. We will capture barriers to recruitment, retention and study completion as well as identify facilitators for successful engagement of nurses and patients. We will record any adverse events to capture safety of the intervention. Participants will have a support from trained dialysis nurses and the Research Officer throughout the study and will be regularly asked about adverse effects as part of the standardised weekly review with the research officer who will be trained to conduct the study. If a patient reports adverse effects, the research officer will discuss this with the participant and with permission, members of the study team. The study team comprises medical and psychology-trained members who will determine the appropriate course of action. The treating nephrologist or the renal psychologist (INJECT investigator) will be involved as appropriate. Finally, we will evaluate clinical outcomes with respect to dialysis vascular access. As this is a feasibility pilot, the assessment of validity or reliability is outside the scope of this study and it will not be assessed.

**Table 4:** Outcome measures

Outcome domain	Evaluation measures
Primary outcome	
Patient feasibility/acceptability of the intervention	• Patient INJECT evaluation survey: evaluates the feasibility and acceptance of the intervention, identifies which components were most useful or desired (Additional file 4). Self-devised survey uses a 5-point scale (1 = very unsuccessful, 5 = very successful and 1 = not at all, 5 = a lot). A higher score indicates higher success/acceptance of the intervention. • INJECT study satisfaction survey: assesses satisfaction with the content of the online intervention and the ability to understand and utilise presented learning material (Additional file 5). Self-devised survey uses a 5-point scale (1 = disagree, 5 = agree). A higher score indicates greater satisfaction. • Engagement measures: (i) The reach (i.e. proportion of participants who were approached who then agreed to take part in the study) (ii) Retention rates (iii) Completion rates (iv) Online module metrics and analytics—average time spent on page, time to completion of modules, engagement with comments section of platform (v) Reporting to project officer during the study period Semi-structured interviews with study participants to describe their experiences and preferences
Secondary outcomes	
Measures of needle distress	• Blood/Injection Fear Scale (BIFS) obtains specific information on physiological symptoms of anxiety [33]. The scale measures fear of blood/injection on a 5-point scale (1 = strongly agree, 5 = strongly disagree). Lower score indicates higher level of fear. Chronic disease patients’ data for reliability and validity: Cronbach’s α 0.98 • Managing Needle Distress Questionnaire (MNDQ), a self-devised questionnaire to assess patient’s distress (Additional file 6). Self-devised unvalidated survey measures distress on a 5-point scale (1 = agree, 5 = disagree). A higher score indicates greater distress. • Dialysis Fear of Injection Questionnaire (DFIQ) assesses a change in managing needle distress [9]. It measures fear of injection on a 4-point scale (0 = almost never, 3 = almost always). Renal data for reliability and validity: sensitivity 0.88, specificity 0.72, Cronbach’s α 0.87.
Measure of distress	Hospital Anxiety & Depression Scale (HADS), a standardised tool for measuring anxiety and depression [34]. The scale consists of 2 subscales: anxiety and depression. Each subscale has 7 items with a score of 0 to 21. A higher score indicates greater anxiety/depression. Renal data for reliability and validity: sensitivity 0.81, specificity 0.90, Cronbach’s α 0.85.
Cannulation outcomes (vascular access/clinical outcomes)	• Collected from the hospital electronic system as - Missed cannulations—i.e. missed veins in the initial attempt or subsequent attempt(s) of needling (at each dialysis session for the duration of the study) - Access surgical interventions (at each dialysis session for the duration of the study)
Acceptability and evaluation of nursing education program	Surveys administered before and after the education to evaluate self-rated knowledge about cannulation practices, confidence in managing patients with needle distress, and ability to support patients participating in INJECT. Self-devised unvalidated survey with scores 1 (disagree) to 5 (agree). Higher score indicates greater knowledge/confidence/abilities. Semi-structured interviews with dialysis nurses to describe their experiences and preferences

### Progression criteria

Decision-making about progression to a larger scale evaluation will be based on a process evaluation of recruitment, retention, intervention acceptability and feasibility [37]. Process evaluation will occur at regular intervals throughout the pilot study.

- If we recruit between 50 and 80% of the target sample, the recruitment strategy will be reviewed to determine the number of patients approached and screened, the number of patients that met eligibility criteria and the number of eligible patients agreeing to participate. Based on the findings, we will establish whether to proceed with a larger evaluation with some minor modifications.
- If less than 50% of participants are retained in the study, a list of reasons for attrition will be inspected and progression to a larger trial will be reconsidered and the potential intervention reviewed and refined.
- Outcome data for at least 80% of participants who have completed online education program will be required for the progression to a larger trial.
- The patient evaluation survey together with exit interviews with patients will provide feedback on each component of the intervention. If the majority of patients find an individual component of the intervention useful (by selecting either agree or strongly agree in the survey), that component will be retained. A lower score will help inform the decision whether some modifications are required, or whether it is realistic to proceed with a component of the intervention.
- Feedback from qualitative interviews will also assist in optimising the intervention.

These progression criteria will inform the decision about trial conduct and review trial processes in order to decide whether the continuation to the main trial is appropriate.

### Planned statistical analysis

Descriptive statistics will be employed to describe the cohort demographic and to analyse survey responses. Qualitative survey data (i.e. responses to open-ended questions) will be extracted using narrative synthesis. Reasons for ineligibility and for non-participation will be reported. Data collected from the DFIQ, MNDQ, BIFS and HADS questionnaires will be analysed by Wilcoxon matched pairs signed-rank tests (Stata software) to calculate changes in scores over the study data collection time points.

### Dissemination

Results of the study will be made available to participants via a lay summary which will be co-designed with consumer partners. Results of the study will be published in peer-reviewed journals, shared via conference presentations and made publicly available via consumer networks.

### Patients as research partners

HD patients have been involved at all stages of the project including in co-investigator roles, in the intervention design and development process and for ongoing input throughout the duration of the study. The patient co-investigator and author who has lived experience as a dialysis and kidney transplant patient has been integrally involved in the research process.

Patients’ input from qualitative interviews preceding this study informed the INJECT intervention. The online modules were co-designed with 3 HD patients. The consumer-centred design ensures that the project is respectful of and responsive to the preferences, needs and values of patients. This project is a major step forward for people trying to live well on dialysis—and may also help patients with other diseases where needles are frequently required.

## Discussion

This study benefits from several strengths. It is a pragmatically designed intervention, based on the established CBT approaches which are considered a gold standard treatment for fear and anxiety disorders. To our knowledge, none have been validated for needle distress in dialysis patients or renal services thus far. The design of the intervention is flexible and adaptable, and it does not require face-to-face contact allowing it to be readily translated to rural and remote settings. The study is underpinned by consumer voices and integration of patients’ needs at all levels of the intervention. A further strength is the ability of nurses to recognise and validate patients’ distress and support the use of strategies they have learned in the study to improve care at the time of cannulation. There are also several limitations to this study. The inclusion criteria require a moderate level of computer and health literacy for participation and therefore may not be suited to all patients. The intervention currently is not specifically designed for culturally and linguistically diverse populations, and expansion into these groups would require specific co-design with patients from those groups to adapt and develop the intervention further. Finally, INJECT is based upon a high level of patient activation and nursing engagement to deliver and complete the intervention. However, we believe many patients with needle distress will be motivated to undertake the intervention due to the lack of any other tools at their disposal to manage needle fear. This is supported by findings from the scoping review performed by our group which revealed a lack of initiatives to address needle fear in adults living with chronic disease [10]. Qualitative study we performed with nurses and patients (to be published separately) revealed that patients feel powerless, unsupported and mostly left to manage alone. Consumer partners on this project voiced their strong support for the multifaceted intervention designed for dialysis patients who experience fear of needles. In addition, extensive national workshops conducted as a part of the Better Evidence and Translation in chronic kidney disease program addressed patient recruitment and retention in clinical trials in chronic kidney disease [38]. Patients were motivated to participate in clinical trials if they trusted the clinicians, if the format was easy to understand, if results are communicated and burden of participation minimised [38]. These findings were considered when designing the INJECT intervention.

Active management of needle distress in HD patients remains a challenge, due to paucity of research support in this area. The INJECT intervention is an important step forward in addressing this common and serious issue. The results of this pilot study will inform the further refinement of each element of the INJECT intervention. Future trials addressing needle distress using some or all of the intervention components included in this pilot study, depending on their feasibility and acceptability, will be a major advance for patients living with haemodialysis. If successful, this intervention is likely to be useful in other chronic disease cohorts such as patients with cancer, diabetes and chronic immune conditions that also have excessive exposure to intravenous cannulation.

## Supplementary Information

**Additional file 1.** The Standard Protocol Items Recommendations for Trials (SPIRIT) checklist.

**Additional file 2.** CONSORT 2010 checklist of information to include when reporting a pilot or feasibility trial.

**Additional file 3.** Patient Demographic Questionnaire.

**Additional file 4.** Patient INJECT evaluation survey.

**Additional file 5.** INJECT Study satisfaction survey.

## Data Availability

No data is available.

## Abbreviations

HD: Haemodialysis
DFIQ: Dialysis Fear of Injection Questionnaire
CBT: Cognitive behavioural therapy
VR: Virtual reality
MNDQ: Managing Needle Distress Questionnaire
BIFS: Blood Injection Fear Scale
HADS: Hospital Anxiety & Depression Scale
