# Supplementary material for: Improving management of needle distress during the journey to dialysis through psychological education and training—the INJECT study feasibility pilot protocol
Source: Pilot Feasibility Stud. 2022 Feb 4;8:28. doi: 10.1186/s40814-022-00989-2 (PMC8815234; doi:10.1186/s40814-022-00989-2)
Supplement: Supplementary file 3 — Additional file 3. Patient Demographic Questionnaire. [file 40814_2022_989_MOESM3_ESM.docx]

**Patient Demographic Questionnaire**

| Name: Study ID number: | Age: |  |
| --- | --- | --- |
| Gender:   - Male - Female - Other:   Current living situation   - Live alone - Live with a partner - Live with family members (children, relatives etc) - Live in a residential facility - Other: | Previous kidney replacement therapy(ies)   - Hospital/satellite haemodialysis - Home haemodialysis - Automated peritoneal dialysis - Continuous ambulatory peritoneal dialysis - Kidney transplant |  |
| Ethnicity:   - Caucasian - Aboriginal/Torres Strait Islander - Other   Occupational status:   - Unemployed (due to kidney disease) - Unemployed (other) - Disability pension - Part-time/casual - Full-time - Retired - Student - Other: | Previous access type for haemodialysis:   - AV fistula - AV Graft - Permacath |  |
|  |  |  |
| Mental Health  Current or past diagnosis of needle phobia?   - Yes - No - Unsure   Are you currently receiving, or have you received professional support for anxiety or distress associated with needles?   - Yes - No - Unsure   Are you currently receiving, or have you received professional support for other mental health concern(s)?   - Yes - No - Unsure   Have you ever received cognitive behavioural therapy?   - Yes - No - Unsure   Are you currently taking any medications for any mental health condition(s)?   - Yes - No - Unsure | | |
