# Supplementary material for: Improving management of needle distress during the journey to dialysis through psychological education and training—the INJECT study feasibility pilot protocol
Source: Pilot Feasibility Stud. 2022 Feb 4;8:28. doi: 10.1186/s40814-022-00989-2 (PMC8815234; doi:10.1186/s40814-022-00989-2)
Supplement: Supplementary file 4 — Additional file 4. Patient INJECT evaluation survey. [file 40814_2022_989_MOESM4_ESM.docx]

**Patient – INJECT Evaluation Survey (feasibility and acceptability)**

(to be completed at the end of 6-week intervention)

Please evaluate the intervention and its separate components by selecting a box which best describes your agreement or disagreement with each statement. Please place a tick only in one box for each statement.

|  | Strongly disagree (1) | Disagree (2) | Neither agree nor disagree (3) | Agree (4) | Strongly agree (5) |  |
| --- | --- | --- | --- | --- | --- | --- |
| 1. INJECT intervention helped me better manage needle distress |  |  |  |  |  |  |
| 2. Online education modules were helpful |  |  |  |  |  |  |
| 3. Psychologist review was helpful |  |  |  |  |  |  |
| 4. Project officer checking in was helpful |  |  |  |  |  |  |
| 5. Nurse support was helpful |  |  |  |  |  |  |
| 6. Virtual reality was helpful (if you haven’t used it please select Not used) |  |  |  |  |  | Not used |
| 7. I would recommend the intervention to someone else |  |  |  |  |  |  |

Any other comments about your participation in this study? (e.g. your experience at the psychology consult, the distress management modules, virtual reality technology or anything else) ____________________________________________________________________________________________________________________________________________________________________________________________________________________________________________________________________________________________________________________________________________________________________________________________
