# Supplementary material for: Improving management of needle distress during the journey to dialysis through psychological education and training—the INJECT study feasibility pilot protocol
Source: Pilot Feasibility Stud. 2022 Feb 4;8:28. doi: 10.1186/s40814-022-00989-2 (PMC8815234; doi:10.1186/s40814-022-00989-2)
Supplement: Supplementary file 5 — Additional file 5. INJECT Study satisfaction survey. [file 40814_2022_989_MOESM5_ESM.docx]

**INJECT Study Satisfaction Survey**

(to be completed immediately after the completion of online modules)

1. The content of the modules was relevant to me

| 1. Disagree | 1. Somewhat disagree | 1. Not sure | 1. Somewhat agree | 1. Agree |
| --- | --- | --- | --- | --- |

1. I understood the content of the modules

| 1. Disagree | 1. Somewhat disagree | 1. Not sure | 1. Somewhat agree | 1. Agree |
| --- | --- | --- | --- | --- |

1. I will continue to apply what I learned from the modules to effectively manage my distress

| 1. Disagree | 1. Somewhat disagree | 1. Not sure | 1. Somewhat agree | 1. Agree |
| --- | --- | --- | --- | --- |

If not, please explain why not: ___________________________________________ ___________________________________________________________________

1. Overall, the modules were useful

| 1. Disagree | 1. Somewhat disagree | 1. Not sure | 1. Somewhat agree | 1. Agree |
| --- | --- | --- | --- | --- |

1. I require further support and information on using strategies to manage needle distress

| 1. Disagree | 1. Somewhat disagree | 1. Not sure | 1. Somewhat agree | 1. Agree |
| --- | --- | --- | --- | --- |

1. Please tick the main strategies that you expect to continue to use to help you manage distress associated with dialysis needles

- Relaxed breathing
- Visual imagery
- Awareness and observation of thoughts (mindfulness)
- Thought challenging
- Developing positive coping strategies
- Acceptance of distress
- Distraction
- I would like to continue using virtual reality technology

1. Any other comments/feedback about the modules:___________________________

___________________________________________________________________
